# RLNSF-MDA: reliability-guided graph-regularized matrix factorization for immune-related miRNA–disease association prediction

**DOI:** 10.3389/fbinf.2026.1926188

**Published:** 2026-08-13

**Authors:** Xin Li, Yaoyu Liu, Ming Xu

**Affiliations:** 1 Department of Immunology, School of Basic Medical Science, Central South University, Changsha, Hunan, China; 2 School of Computer Science, The University of Sydney, Sydney, NSW, Australia

**Keywords:** Gaussian interaction profile, graph-regularized matrix factorization, immune-related disease, miRNA–disease association, reliability-guided similarity fusion

## Abstract

**Introduction:**

MicroRNAs (miRNAs) regulate gene expression and are closely linked to the onset and progression of immune‑related diseases. Experimental discovery of disease‑associated miRNAs remains costly and time‑consuming, motivating computational prioritization. However, existing matrix‑completion and deep graph‑learning methods either underuse local biological neighborhood evidence or require complex multi‑view neural architectures.

**Methods:**

In this study, we present RLNSF‑MDA, a reliability‑guided graph‑regularized logistic matrix factorization framework for predicting potential miRNA–disease associations, with an emphasis on immune disease analysis. The method integrates disease semantic similarity; miRNA functional, semantic, and sequence similarities; and training‑only Gaussian interaction profile similarities through data‑driven reliability weights. It then combines multi‑scale neighborhood evidence, diffusion scores, low‑rank reconstruction features, and contrastive graph‑regularized latent factors.

**Results:**

On the HMDD v3.2 benchmark containing 788 miRNAs and 374 diseases, RLNSF‑MDA achieved an average accuracy of 0.8525, an AUC of 0.9194, and an AUPR of 0.9055 in five‑fold cross‑validation, and an average accuracy of 0.8569, an AUC of 0.9266, and an AUPR of 0.9197 in ten‑fold cross‑validation. Ablation experiments showed that the full model outperformed reduced feature combinations and training strategies, supporting the contribution of reliability‑guided fusion, side‑score construction, graph regularization, and contrastive ranking. Evidence from immune‑related case studies in dbDEMC V2.0 and miR2Disease further covered lupus nephritis, lymphoma, and leukemia, with 45, 47, and 47 confirmed miRNAs, respectively.

**Discussion:**

These results suggest that RLNSF‑MDA provides an effective and interpretable framework for prioritizing candidate miRNAs associated with immune‑related diseases.

## 1 Introduction

MicroRNAs (miRNAs) are short non-coding RNAs that regulate gene expression after transcription and can influence many biological processes through coordinated effects on multiple targets (Bartel, 2004; Bartel, 2009). In immune-disease research, miRNAs have been linked to lupus nephritis, lymphoma, leukemia, psoriasis, multiple sclerosis, and inflammatory bowel disease, making them relevant both as molecular regulators and as potential biomarkers (Huang et al., 2019b; Jiang et al., 2009; Yang et al., 2017). Database and translational evidence have also connected specific miRNAs with immune-mediated tissue remodeling, disease-enriched miRNA profiles, and experimentally reported immune–disease associations. These findings motivate systematic identification of miRNA–disease associations, especially for immune-related diseases where early risk stratification and mechanistic interpretation remain important.

Experimentally verifying miRNA–disease associations is informative but expensive, labor-intensive, and incomplete relative to the number of possible miRNA–disease pairs. Curated resources such as HMDD and miR2Disease have, therefore, become important foundations for computational prioritization by collecting experimentally supported or manually curated disease-related miRNA evidence (Huang et al., 2019b; Jiang et al., 2009). Additional resources, including dbDEMC for differentially expressed miRNAs, provide complementary evidence for external validation in disease case studies (Yang et al., 2017). These databases enable miRNA–disease association prediction to be treated as a supervised or semi-supervised prioritization problem: given a sparse known association matrix and biological similarity information, the goal is to assign high scores to plausible but unverified miRNA–disease links.

Many computational models have been proposed for this task. Network-based approaches use the topology of miRNA-disease graphs or heterogeneous biological networks to propagate association evidence, as illustrated by KATZMDA, WBSMDA, HGIMDA, RLSMDA and RKNNMDA (Qu et al., 2018; Ji et al., 2026; Ji et al., 2020a; Ji et al., 2020b; Chen et al., 2017). Matrix-completion and matrix-factorization approaches instead exploit low-rank structure in the association matrix, with representative methods including MCMDA, MDHGI, graph-regularized non-negative matrix factorization, inductive matrix completion and collaborative matrix factorization (Li et al., 2017; Xiao et al., 2018; Shen et al., 2017). More recent multi-view neural models combine graph representation learning with matrix factorization to learn richer latent features (Ding et al., 2022; Ji et al., 2024). However, independent benchmarking has shown that prediction performance and robustness can vary substantially across datasets and settings, and that available predictors may be biased toward diseases with many known associations (Huang et al., 2019a).

This variation points to a practical challenge for immune-related miRNA prioritization. Useful predictors should integrate multiple biological similarity sources, avoid test-set leakage when constructing association-derived similarities, preserve interpretable local evidence from similar miRNAs and diseases, and remain reproducible without relying on overly complex multi-view neural architectures. Existing graph and matrix-factorization methods address parts of this problem, but they often treat similarity sources with fixed weights, emphasize either global topology or latent low-rank structure, or provide limited separation between local neighborhood evidence and the final scoring model. For sparse immune disease entries, this can make it difficult to determine whether a candidate is supported by consistent biological neighborhoods, global propagation, or only by dataset popularity.

**FIGURE 1:**
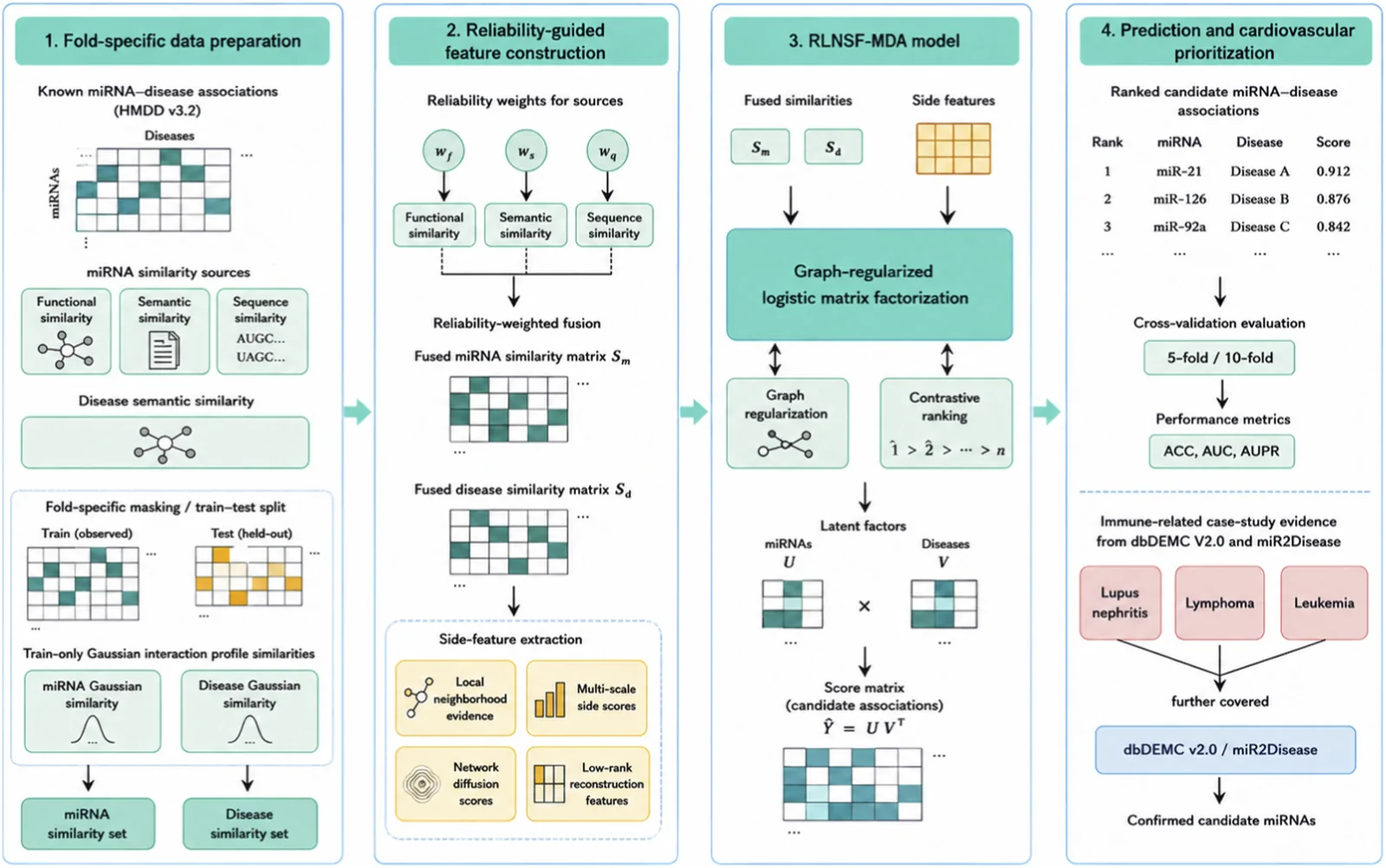
Overview of RLNSF-MDA. The framework performs fold-specific data preparation using known miRNA–disease associations and multiple biological similarity sources. Reliability-guided fusion is used to construct robust miRNA and disease similarity matrices, from which neighborhood, diffusion, multi-scale, and low-rank side features are extracted. These features are integrated into a graph-regularized logistic matrix factorization model with contrastive ranking to score candidate miRNA–disease associations. The predicted associations are evaluated by cross-validation and further examined in immune-related disease case studies using external evidence from dbDEMC v2.0 and miR2Disease.

To address these issues, we propose RLNSF-MDA, a reliability-guided graph-regularized logistic matrix factorization framework for miRNA-disease association prediction. The method constructs train-only Gaussian interaction profile similarities to reduce information leakage, assigns data-driven reliability weights to disease and miRNA similarity sources, extracts multi-scale neighborhood and diffusion evidence, and combines these side scores with graph-regularized latent factors and a contrastive ranking objective. This design aims to retain the interpretability of similarity-based prediction while using trainable matrix factorization to capture global association structure. Figure 1 summarizes the workflow from fold-specific masking and similarity fusion to calibrated candidate prioritization. We evaluate RLNSF-MDA on the HMDD v3.2 benchmark and further examine immune-related case-study evidence for lupus nephritis, lymphoma, and leukemia, focusing on whether the model can provide accurate and biologically traceable candidate prioritization.

## 2 Methods

The Methods section follows the modular style used in recent matrix-completion and multi-view miRNA-disease association studies, but the proposed model differs from AMCMDA and MUSCLE in its core design: AMCMDA recovers a heterogeneous association matrix through truncated nuclear norm minimization, whereas MUSCLE uses multi-view graph attention and multi-scale feature fusion (Li et al., 2025; Ji et al., 2024). RLNSF-MDA instead combines reliability-guided similarity fusion, interpretable side evidence, and graph-regularized logistic matrix factorization. The complete workflow contains seven steps: (i) task formulation and fold-specific masking, (ii) train-only Gaussian interaction profile construction, (iii) reliability-guided similarity fusion, (iv) multi-scale local and propagation feature construction, (v) contrastive graph-regularized logistic matrix factorization, (vi) score calibration, and (vii) cross-validation and case-study evaluation.

### 2.1 Task formulation and data representation

Let $M=\left\{m_{1},\ldots ,m_{n_{m}}\right\}$ denote the set of miRNAs and $D=\left\{d_{1},\ldots ,d_{n_{d}}\right\}$ denote the set of diseases. The known miRNA–disease association matrix is defined as Equation 1

$$A_{ij}=\left\{\begin{array}{c} 1,\text{if miRNA }m_{i}\text{ is experimentally associated with }\\ \text{disease }d_{j},\\ 0,\text{ otherwise}, \end{array}\right.\;A\in {\left\{0,1\right\}}^{n_{m}\times n_{d}}.$$ (1)

In this study, the HMDD v3.2 benchmark contains $n_{m}=788$ miRNAs and $n_{d}=374$ diseases. We used one disease semantic similarity matrix and three miRNA similarity matrices, namely, miRNA functional similarity, miRNA semantic similarity, and miRNA sequence similarity. For each cross-validation split, test positive associations were first removed from A to obtain a fold-specific training matrix $A^{\text{tr}}$ . All association-derived similarities and side features were then computed from $A^{\text{tr}}$ only so that hidden test associations were not used during feature construction.

### 2.2 Train-only Gaussian interaction profile similarities

Gaussian interaction profile (GIP) kernels convert association profiles into similarity matrices by assuming that nodes with similar interaction profiles are likely to share biological functions (van Laarhoven et al., 2011). For miRNA $m_{i}$ , the training interaction profile is the i th row of the masked matrix, $g_{m}\left(i\right)=A_{i,:}^{\text{tr}}$ . The miRNA GIP similarity is Equation 2:

$$S_{m}^{\text{GIP}}\left(i,l\right)=\exp\left(-\gamma_{m}{\left\|g_{m}\left(i\right)-g_{m}\left(l\right)\right\|}_{2}^{2}\right),\;\gamma_{m}=\frac{n_{m}}{\sum_{i=1}^{n_{m}}{\left\|g_{m}\left(i\right)\right\|}_{2}^{2}}.$$ (2)

For disease $d_{j}$ , the training interaction profile is the j th column, $g_{d}\left(j\right)=A_{:,j}^{\text{tr}}$ . The disease GIP similarity is Equation 3:

$$S_{d}^{\text{GIP}}\left(j,l\right)=\exp\left(-\gamma_{d}{\left\|g_{d}\left(j\right)-g_{d}\left(l\right)\right\|}_{2}^{2}\right),\;\gamma_{d}=\frac{n_{d}}{\sum_{j=1}^{n_{d}}{\left\|g_{d}\left(j\right)\right\|}_{2}^{2}}.$$ (3)

The diagonal values of each GIP matrix were set to 1. When nearest neighbors were selected, the diagonal was temporarily ignored to prevent a node from selecting itself.

### 2.3 Reliability-guided multi-source similarity fusion

The method assigns a reliability weight to each similarity source according to whether its nearest neighbors have consistent training association profiles. For a similarity source $S^{\left(r\right)}$ , let $N_{K}\left(u;S^{\left(r\right)}\right)$ be the top- K neighbors of node u under $S^{\left(r\right)}$ , excluding u itself. Let $p_{u}$ be the training association profile of node u and Equation 4:

$$c\left(u,v\right)=\frac{p_{u}^{⊤}p_{v}}{\max\left({\left\|p_{u}\right\|}_{2}{\left\|p_{v}\right\|}_{2},\epsilon \right)}$$ (4)

be the cosine similarity between two association profiles, with ϵ preventing division by zero. The reliability score of source r is Equation 5:

$$\rho_{r}=\max\left(\epsilon ,\frac{1}{n}\overset{n}{\underset{u=1}{\sum }}\frac{1}{K}\underset{v\in N_{K}\left(u;S^{\left(r\right)}\right)}{\sum }c\left(u,v\right)\right),$$ (5)

and the normalized source weight is Equation 6:

$$w_{r}=\frac{\rho_{r}}{\sum_{q}\rho_{q}}.$$ (6)

In the experiments, K=10 . The fused disease similarity matrix uses disease semantic similarity $S_{d}^{\text{sem}}$ and train-only disease GIP similarity is Equation 7:

$$S_{d}=w_{d}^{\text{sem}}S_{d}^{\text{sem}}+w_{d}^{\text{GIP}}S_{d}^{\text{GIP}}.$$ (7)

The fused miRNA similarity matrix integrates functional, semantic, sequence and train-only GIP similarities is Equation 8:

$$S_{m}=w_{m}^{\text{fun}}S_{m}^{\text{fun}}+w_{m}^{\text{sem}}S_{m}^{\text{sem}}+w_{m}^{\text{seq}}S_{m}^{\text{seq}}+w_{m}^{\text{GIP}}S_{m}^{\text{GIP}}.$$ (8)

This reliability weighting keeps similarity sources that agree with the training association structure and down-weights sources whose nearest-neighbor structure is less consistent with known associations.

### 2.4 Multi-scale local and propagation features

For a candidate pair $\left(m_{i},d_{j}\right)$ , RLNSF-MDA constructs interpretable side features at neighborhood scales k∈{5,10,20} . Let $N_{k}^{d}\left(j\right)$ be the top- k disease neighbors of $d_{j}$ under $S_{d}$ and $N_{k}^{m}\left(i\right)$ be the top- k miRNA neighbors of $m_{i}$ under $S_{m}$ . The disease-side and miRNA-side local evidence scores are Equation 9:

$$e_{d,k}\left(i,j\right)=\frac{\sum_{l\in N_{k}^{d}\left(j\right)}S_{d}\left(j,l\right)A_{i,l}^{\text{tr}}}{\sum_{l\in N_{k}^{d}\left(j\right)}S_{d}\left(j,l\right)+\epsilon },\;e_{m,k}\left(i,j\right)=\frac{\sum_{l\in N_{k}^{m}\left(i\right)}S_{m}\left(i,l\right)A_{l,j}^{\text{tr}}}{\sum_{l\in N_{k}^{m}\left(i\right)}S_{m}\left(i,l\right)+\epsilon }.$$ (9)

The fused local neighborhood score is Equation 10

$$e_{k}^{\text{local}}\left(i,j\right)=\frac{e_{d,k}\left(i,j\right)+e_{m,k}\left(i,j\right)}{2}.$$ (10)

The same equations are also computed on the GIP attribute graphs to provide GIP-specific local evidence. To capture global propagation through the fused similarity networks, row-normalized matrices ${\hat{S}}_{m}$ and ${\hat{S}}_{d}$ are used to define Equation 11:

$$D_{1}={\hat{S}}_{m}A^{\text{tr}},\;D_{2}=A^{\text{tr}}{\hat{S}}_{d},\;D_{3}={\hat{S}}_{m}A^{\text{tr}}{\hat{S}}_{d}.$$ (11)

An additional second-order propagation matrix is Equation 12:

$$D_{4}={\hat{S}}_{m}D_{3}{\hat{S}}_{d},$$ (12)

and the propagation ensemble score is Equation 13:

$$P=0.20D_{1}+0.20D_{2}+0.35D_{3}+0.25D_{4}.$$ (13)

Low-rank side scores are constructed by singular value decomposition of $A^{\text{tr}}$ at ranks 16, 32, 64, and 128. A heterogeneous matrix is also formed as Equation 14:

$$H=\left[\begin{array}{cc} {\hat{S}}_{m} & A^{\text{tr}}\\ {\left(A^{\text{tr}}\right)}^{⊤} & {\hat{S}}_{d} \end{array}\right],$$ (14)

and its reconstructed off-diagonal block is used as a heterogeneous low-rank score, similar in spirit to heterogeneous network construction in matrix-completion methods (Li et al., 2025). The final side-feature vector $x_{ij}$ concatenates multi-scale local scores, GIP-local scores, diffusion scores, propagation scores, SVD reconstruction scores, mean top-20 similarities, and node degrees.

### 2.5 Contrastive graph-regularized logistic matrix factorization

The latent miRNA and disease embeddings are initialized from spectral decompositions of the fused similarity graphs. If ${\hat{S}}_{m}\approx Q_{m}\Lambda_{m}Q_{m}^{⊤}$ and ${\hat{S}}_{d}\approx Q_{d}\Lambda_{d}Q_{d}^{⊤}$ , the initial embedding matrices are Equation 15:

$$U^{\left(0\right)}=Q_{m}\left[:,1:E\right]\Lambda_{m}{\left[:,1:E\right]}^{1/2},\;V^{\left(0\right)}=Q_{d}\left[:,1:E\right]\Lambda_{d}{\left[:,1:E\right]}^{1/2},$$ (15)

where E is the latent dimension. Parameter analysis selected E=128 for the reported model. For pair $\left(m_{i},d_{j}\right)$ , the matrix-factorization logit is Equation 16:

$$z_{ij}=u_{i}^{⊤}v_{j}+b_{i}+c_{j},\;{\hat{y}}_{ij}=\sigma \left(z_{ij}\right)=\frac{1}{1+\exp\left(-z_{ij}\right)},$$ (16)

where $u_{i}$ and $v_{j}$ are trainable embeddings and $b_{i},c_{j}$ are trainable bias terms. The binary cross-entropy loss over sampled training pairs T is Equation 17:

$$L_{\text{BCE}}=-\frac{1}{\left|T\right|}\underset{\left(i,j\right)\in T}{\sum }\left[y_{ij}\log{\hat{y}}_{ij}+\left(1-y_{ij}\right)\log\left(1-{\hat{y}}_{ij}\right)\right].$$ (17)

To encourage verified associations to rank above sampled unknown pairs, we add a Bayesian personalized ranking-style contrastive loss is Equation 18:

$$L_{\text{rank}}=-\frac{1}{\left|R\right|}\underset{\left(\left(i,j^{+}\right),\left(a,b^{-}\right)\right)\in R}{\sum }\log\sigma \left(z_{ij^{+}}-z_{ab^{-}}\right),$$ (18)

where $\left(i,j^{+}\right)$ is a known positive pair and $\left(a,b^{-}\right)$ is a sampled unknown pair. Graph smoothness is imposed on top-20 similarity edges from the fused miRNA and disease graphs is Equation 19:

$$L_{\text{graph}}=\frac{1}{\left|E_{m}\right|}\underset{\left(i,l\right)\in E_{m}}{\sum }S_{m}\left(i,l\right){\left\|u_{i}-u_{l}\right\|}_{2}^{2}+\frac{1}{\left|E_{d}\right|}\underset{\left(j,l\right)\in E_{d}}{\sum }S_{d}\left(j,l\right){\left\|v_{j}-v_{l}\right\|}_{2}^{2}.$$ (19)

The total optimization objective is Equation 20:

$$L=L_{\text{BCE}}+\lambda_{r}L_{\text{rank}}+\lambda_{g}L_{\text{graph}}+\lambda_{2}{\left\|\Theta \right\|}_{2}^{2}.$$ (20)

The reported model used $\lambda_{r}=0.35$ , $\lambda_{g}=0.002$ , AdamW optimization, 260 training epochs, a batch size of 4,096, and five sampled unknown pairs for each positive pair in the ranking component.

### 2.6 Score calibration and final prediction

The matrix-factorization probability ${\hat{y}}_{ij}$ is combined with the side-feature vector $x_{ij}$ and matrix-derived scores $r_{ij}$ to form a calibration vector is Equation 21:

$$z_{ij}^{\text{cal}}=\left[x_{ij},r_{ij},{\hat{y}}_{ij}\right].$$ (21)

A supervised calibrator $f_{\text{cal}}$ is trained on the training pairs to produce a calibrated probability $q_{ij}=f_{\text{cal}}\left(z_{ij}^{\text{cal}}\right)$ . In the ensemble setting, the final score is Equation 22:

$$s_{ij}=0.92q_{ij}+0.06{\tilde{P}}_{ij}+0.02{\tilde{H}}_{ij},$$ (22)

where ${\tilde{P}}_{ij}$ and ${\tilde{H}}_{ij}$ are min–max normalized propagation-ensemble and heterogeneous SVD scores for the test fold. This final calibration step allows local evidence, global propagation, and latent-factor evidence to contribute to the reported association score.

### 2.7 Cross-validation, evaluation metrics, and case-study validation

The evaluation follows the 5-fold and 10-fold cross-validation style commonly used in recent miRNA–disease association prediction studies (Ji et al., 2024). Known positive associations were randomly partitioned into K folds. In each fold, one fold of positive associations was hidden as test positives, and the same number of unknown miRNA–disease pairs was sampled as test negatives. Training negatives were sampled from unknown pairs that were neither known positives nor selected as test negatives. For the balanced test set, binary predictions were obtained by ranking all test pairs by $s_{ij}$ and assigning the top $N_{+}$ pairs as positive, where $N_{+}$ is the number of test positives. Continuous scores were used directly to compute AUC and AUPR.

For case study validation, immune-related diseases were evaluated using external evidence tables derived from miR2Disease and dbDEMC 2.0 (Jiang et al., 2009; Yang et al., 2017). The validation pipeline normalized miRNA names by removing arm suffixes such as 3p and 5p, merging star forms, and mapping precursor variants to a common identifier. Evidence records were grouped for lupus nephritis, lymphoma, and leukemia using disease-specific priority and support terms. This procedure produced confirmed immune-related miRNA lists that were used to assess whether the candidate associations recovered by RLNSF-MDA were biologically traceable.

## 3 Results

### 3.1 Cross-validation performance on HMDD v3.2

RLNSF-MDA showed stable predictive performance in both cross-validation settings on the HMDD v3.2 benchmark. In five-fold cross-validation, the model achieved an average accuracy of 0.8525 ± 0.0044, an AUC of 0.9194 ± 0.0058, and an average MCC of 0.7050 ± 0.0088 across the five folds (Table 1). The corresponding fold-level AUC values ranged from 0.9128 to 0.9283, indicating limited variation across partitions. In ten-fold cross-validation, the average accuracy increased to 0.8569 ± 0.0107 and the average AUC reached 0.9266 ± 0.0085, with the best fold attaining an AUC of 0.9425 (Table 2). These results indicate that the model maintained consistent performance when the test partitions were made finer and the evaluation became more demanding.

**TABLE 1:** Five-fold cross-validation results on HMDD v3.2.

Fold	Accuracy	Sensitivity	Specificity	Precision	MCC	AUC
First	0.8534	0.8534	0.8534	0.8534	0.7068	0.9186
Second	0.8484	0.8484	0.8484	0.8484	0.6968	0.9128
Third	0.8528	0.8528	0.8528	0.8528	0.7057	0.9233
Fourth	0.8600	0.8600	0.8600	0.8600	0.7200	0.9283
Fifth	0.8477	0.8477	0.8477	0.8477	0.6955	0.9142
Average	0.8525 ± 0.0044	0.8525 ± 0.0044	0.8525 ± 0.0044	0.8525 ± 0.0044	0.7050 ± 0.0088	0.9194 ± 0.0058

**TABLE 2:** Ten-fold cross-validation results on HMDD v3.2.

Fold	Accuracy	Sensitivity	Specificity	Precision	MCC	AUC
First	0.8573	0.8573	0.8573	0.8573	0.7146	0.9248
Second	0.8473	0.8473	0.8473	0.8473	0.6945	0.9240
Third	0.8696	0.8696	0.8696	0.8696	0.7391	0.9332
Fourth	0.8562	0.8562	0.8562	0.8562	0.7124	0.9322
Fifth	0.8495	0.8495	0.8495	0.8495	0.6990	0.9234
Sixth	0.8473	0.8473	0.8473	0.8473	0.6945	0.9179
Seventh	0.8439	0.8439	0.8439	0.8439	0.6878	0.9109
Eighth	0.8606	0.8606	0.8606	0.8606	0.7213	0.9329
Ninth	0.8571	0.8571	0.8571	0.8571	0.7143	0.9238
Tenth	0.8806	0.8806	0.8806	0.8806	0.7612	0.9425
Average	0.8569 ± 0.0107	0.8569 ± 0.0107	0.8569 ± 0.0107	0.8569 ± 0.0107	0.7139 ± 0.0214	0.9266 ± 0.0085

**FIGURE 2:**
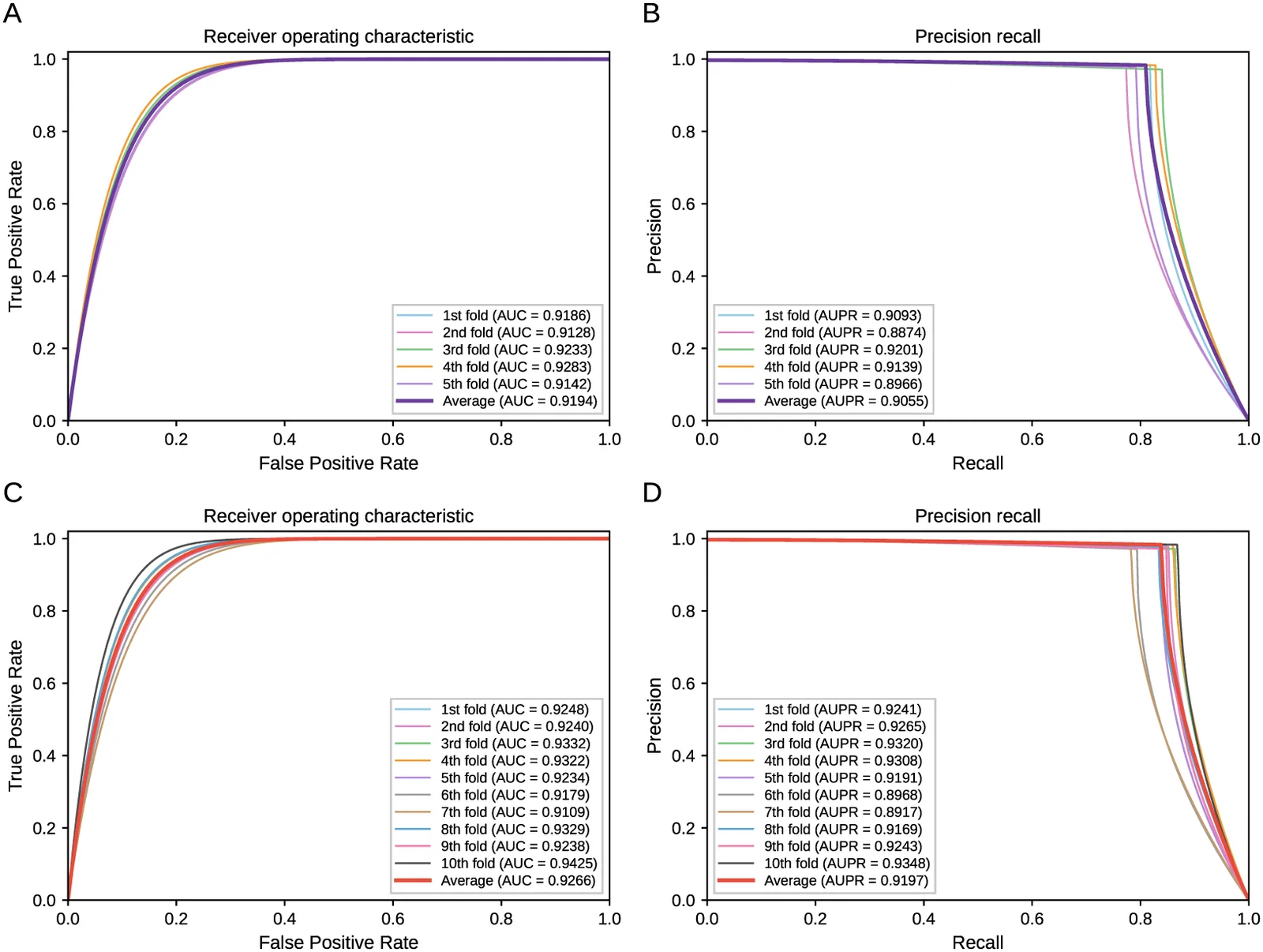
Cross-validation ROC and precision–recall curves for RLNSF-MDA on HMDD v3.2. **(A)** Five-fold ROC curves with fold-wise and average AUC values. **(B)** Five-fold precision–recall curves with fold-wise and average AUPR values. **(C)** Ten-fold ROC curves. **(D)** Ten-fold precision–recall curves.

Table 1 reports the full five-fold results, including sensitivity, specificity, precision and MCC, and Table 2 lists the corresponding ten-fold values. The ROC and precision-recall curves in Figure 2 further support the ranking performance of the method, with average AUC and AUPR values of 0.9194 and 0.9055 in five-fold validation and 0.9266 and 0.9197 in ten-fold validation, respectively.

### 3.2 Parameter sensitivity analysis

We next examined whether the main hyperparameters of RLNSF-MDA affected prediction performance. The embedding dimension controlled the capacity of the latent miRNA and disease factors. As shown in Table 3, increasing the embedding dimension from E=32 to E=128 consistently improved all reported metrics, with AUC increasing from 0.8670 to 0.9194 and AUPR increasing from 0.8457 to 0.9055. Accuracy, precision, recall, and F1-score followed the same trend, increasing from 0.7945 at E=32 to 0.8525 at E=128 . When the embedding dimension was further increased to E=192 , AUC decreased to 0.9139, and AUPR decreased to 0.8983, indicating that E=128 provided the best observed balance in this analysis.

**TABLE 3:** Effect of embedding dimension on RLNSF-MDA performance.

Embedding dimension	AUC	AUPR	Accuracy	Precision	Recall	F1-score
E=32	0.8670	0.8457	0.7945	0.7945	0.7945	0.7945
E=64	0.8909	0.8693	0.8235	0.8235	0.8235	0.8235
E=96	0.9093	0.8928	0.8440	0.8440	0.8440	0.8440
E=128	0.9194	0.9055	0.8525	0.8525	0.8525	0.8525
E=192	0.9139	0.8983	0.8482	0.8482	0.8482	0.8482

**FIGURE 3:**
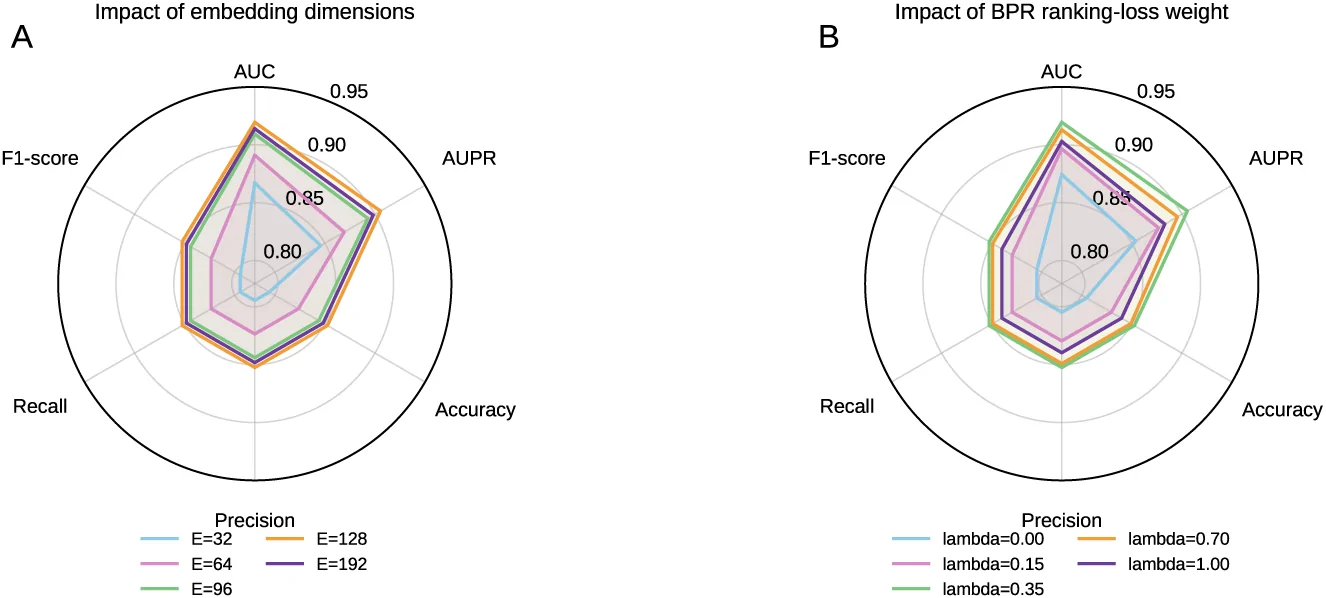
Parameter sensitivity of RLNSF-MDA. **(A)** Effect of the embedding dimension on AUC, AUPR, accuracy, precision, recall, and F1-score. **(B)** Effect of the BPR ranking-loss weight on the same six metrics. The best observed setting in both analyses corresponded to E=128 and λ=0.35 . BPR, Bayesian personalized ranking.

The weight of the Bayesian personalized ranking component also influenced performance. Without the ranking loss (λ=0.00) , RLNSF-MDA achieved an AUC of 0.8743 and an AUPR of 0.8539 (Table 4). Increasing the weight to λ=0.15 improved AUC to 0.8964 and AUPR to 0.8765, and the best results were obtained at λ=0.35 , where AUC, AUPR, and F1-score reached 0.9194, 0.9055, and 0.8525, respectively. Larger weights reduced performance, with AUC values of 0.9130 at λ=0.70 and 0.9029 at λ=1.00 . The radar plots in Figure 3 show the same pattern across AUC, AUPR, accuracy, precision, recall, and F1-score, supporting the use of E=128 and λ=0.35 in the reported model.

**TABLE 4:** Effect of BPR ranking-loss weight on RLNSF-MDA performance.

BPR ranking-loss weight	AUC	AUPR	Accuracy	Precision	Recall	F1-score
λ=0.00	0.8743	0.8539	0.8048	0.8048	0.8048	0.8048
λ=0.15	0.8964	0.8765	0.8295	0.8295	0.8295	0.8295
λ=0.35	0.9194	0.9055	0.8525	0.8525	0.8525	0.8525
λ=0.70	0.9130	0.8955	0.8491	0.8491	0.8491	0.8491
λ=1.00	0.9029	0.8829	0.8397	0.8397	0.8397	0.8397

### 3.3 Ablation of feature combinations and fusion strategies

To isolate the contribution of each design choice, we next evaluated feature combinations and fusion strategies. Starting from the association matrix alone, adding the train-only GIP features improved AUC from 0.8367 to 0.8698 and AUPR from 0.8059 to 0.8430 (Table 5). Incorporating biological similarity further increased AUC to 0.8826, and combining association, biological similarity, GIP, and side scores yielded an AUC of 0.9111 and an AUPR of 0.8937. The full RLNSF-MDA model achieved the best performance, with an AUC of 0.9194, an AUPR of 0.9055, and an accuracy of 0.8525, showing that each module contributed additional predictive value beyond the raw association matrix.

**TABLE 5:** Effect of feature combinations on RLNSF-MDA performance.

Feature	AUC	AUPR	Accuracy	Precision	Recall	F1-score
Assoc	0.8367	0.8059	0.7715	0.7715	0.7715	0.7715
Assoc + GIP	0.8698	0.8430	0.8048	0.8048	0.8048	0.8048
Assoc + BioSim	0.8826	0.8584	0.8175	0.8175	0.8175	0.8175
Assoc + BioSim + GIP	0.8992	0.8783	0.8337	0.8337	0.8337	0.8337
Assoc + BioSim + GIP + Side	0.9111	0.8937	0.8448	0.8448	0.8448	0.8448
RLNSF-MDA	0.9194	0.9055	0.8525	0.8525	0.8525	0.8525

**FIGURE 4:**
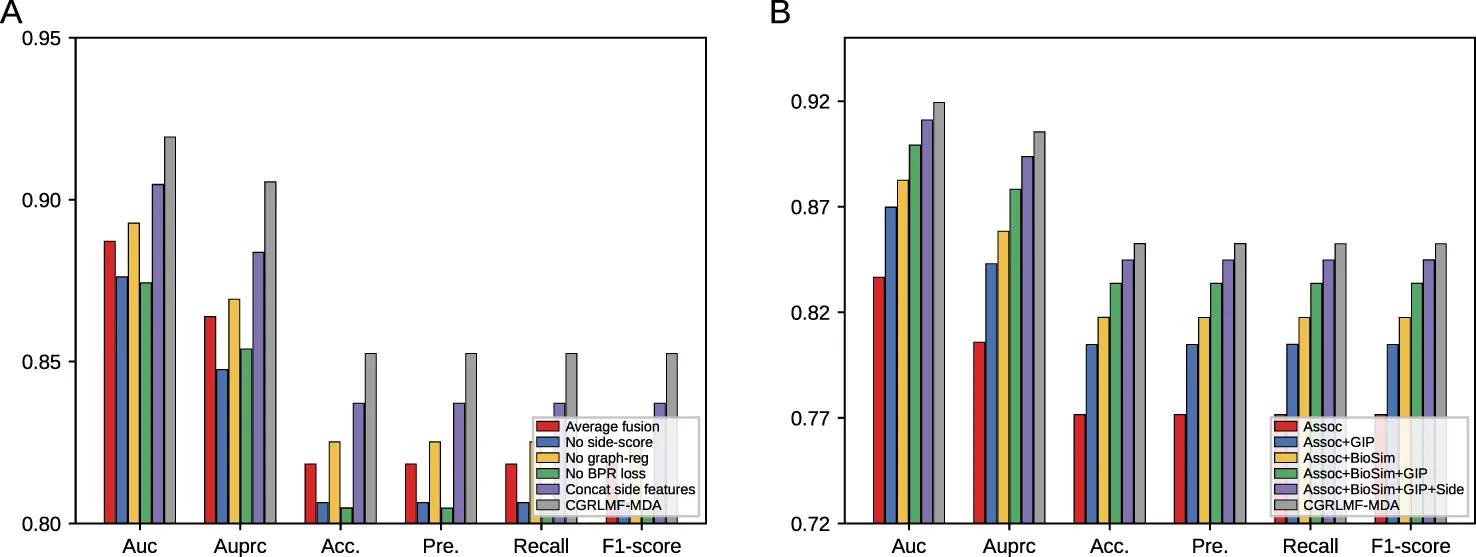
Ablation analysis of RLNSF-MDA. **(A)** Comparison of the effect of progressive feature combinations, from the association matrix alone to the full model, across AUC, AUPR, accuracy, precision, recall, and F1-score. **(B)** Comparison of fusion strategies, including average fusion, removal of side scores, removal of graph regularization, removal of BPR loss, and direct concatenation of side features. The full model outperformed all reduced variants across the reported metrics. BPR, Bayesian personalized ranking; BioSim, biological similarity; GIP, Gaussian interaction profile.

The fusion-strategy ablation provided a complementary view of the same effect. Average fusion produced an AUC of 0.8872 and an AUPR of 0.8638, while removing side scores reduced performance to an AUC of 0.8762 and an AUPR of 0.8475 (Table 6). Disabling graph regularization or the BPR loss also degraded performance, with an AUC decreasing to 0.8927 and 0.8743, respectively. In contrast, concatenating side features improved over simple average fusion, reaching an AUC of 0.9047 and an AUPR of 0.8838, but it remained below the full RLNSF-MDA setting. Figure 4 summarizes these trends across AUC, AUPR, accuracy, precision, recall, and F1-score, indicating that the gains were distributed across both ranking and classification metrics rather than confined to a single score.

**TABLE 6:** Effect of fusion strategies on RLNSF-MDA performance.

Strategy	AUC	AUPR	Accuracy	Precision	Recall	F1-score
Average fusion	0.8872	0.8638	0.8184	0.8184	0.8184	0.8184
No side-score	0.8762	0.8475	0.8065	0.8065	0.8065	0.8065
No graph-reg	0.8927	0.8693	0.8252	0.8252	0.8252	0.8252
No BPR loss	0.8743	0.8539	0.8048	0.8048	0.8048	0.8048
Concatenatingside features	0.9047	0.8838	0.8372	0.8372	0.8372	0.8372
RLNSF-MDA	0.9194	0.9055	0.8525	0.8525	0.8525	0.8525

### 3.4 Comparison with a single-graph strategy

We further compared RLNSF-MDA with a single heterogeneous graph (SHG) strategy to evaluate whether the reliability-guided multi-source design improved upon a simpler graph representation. In five-fold cross-validation, the SHG setting achieved an average accuracy of 0.8218 ± 0.0038, an MCC of 0.6453 ± 0.0075, and an AUC of 0.8974 ± 0.0050 (Table 7). The full RLNSF-MDA model achieved higher values on the same evaluation setting, with an average accuracy of 0.8525 ± 0.0044, an MCC of 0.7050 ± 0.0088, and an AUC of 0.9194 ± 0.0058. The improvement over SHG was, therefore, observed across threshold-based metrics and the ranking metric, rather than only in one evaluation dimension.

**TABLE 7:** Comparison between the single heterogeneous graph strategy and RLNSF-MDA in five-fold cross-validation.

Fold	Accuracy	Sensitivity	Specificity	Precision	MCC	AUC
First	0.8227	0.8244	0.8201	0.8201	0.6467	0.8947
Second	0.8170	0.8187	0.8145	0.8145	0.6369	0.8918
Third	0.8238	0.8221	0.8247	0.8238	0.6478	0.9011
Fourth	0.8273	0.8308	0.8247	0.8256	0.6574	0.9051
Fifth	0.8180	0.8180	0.8180	0.8180	0.6378	0.8941
SHG average	0.8218 ± 0.0038	0.8228 ± 0.0046	0.8204 ± 0.0039	0.8204 ± 0.0040	0.6453 ± 0.0075	0.8974 ± 0.0050
RLNSF-MDA	0.8525 ± 0.0044	0.8525 ± 0.0044	0.8525 ± 0.0044	0.8525 ± 0.0044	0.7050 ± 0.0088	0.9194 ± 0.0058

**FIGURE 5:**
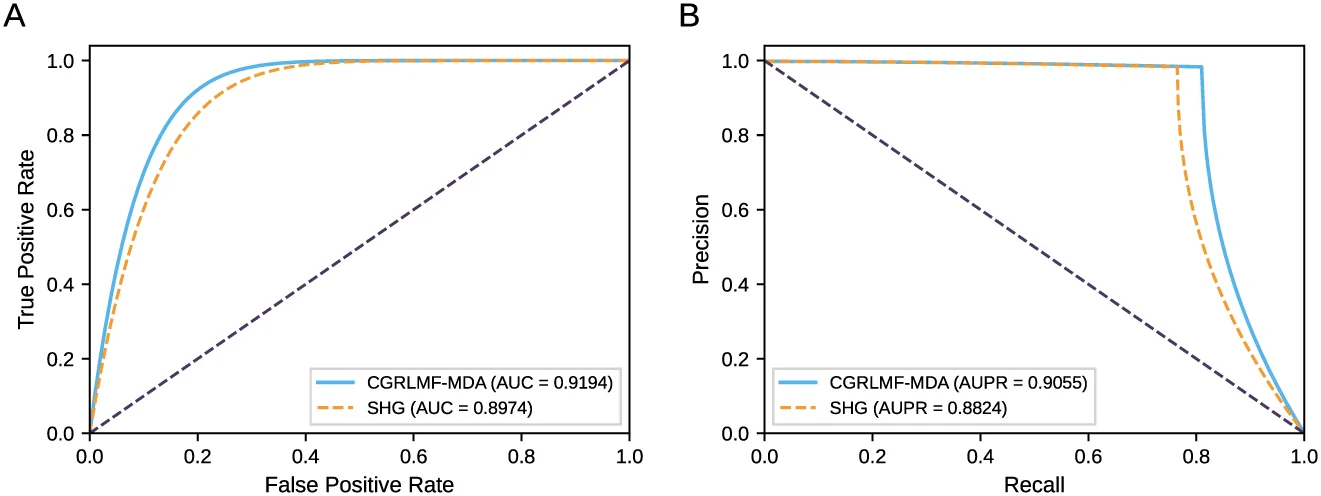
Comparison between RLNSF-MDA and the single heterogeneous graph strategy. **(A)** ROC curves, where RLNSF-MDA achieved an AUC of 0.9194 compared with 0.8974 for SHG. **(B)** Precision–recall curves, where RLNSF-MDA achieved an AUPR of 0.9055 compared with 0.8824 for SHG. SHG, single heterogeneous graph.

The curve-level comparison in Figure 5 confirmed this pattern. RLNSF-MDA produced a higher ROC curve summary than SHG, with an AUC of 0.9194 compared with 0.8974. It also achieved a higher precision–recall summary, with an AUPR of 0.9055 compared with 0.8824. These results indicate that retaining reliability-guided fusion and the full scoring pipeline improved prediction over representing the task with a single heterogeneous graph alone.

### 3.5 Comparison with state-of-the-art miRNA–disease association predictors

Finally, we compared RLNSF-MDA with representative state-of-the-art miRNA–disease association predictors, including KATZMDA, GRNMF, HGIMDA, IMCMDA, RLSMDA, RKNNMDA, and WBSMDA (Qu et al., 2018; Xiao et al., 2018; Chen et al., 2016b; Chen et al., 2018; Chen and Yan, 2014; Chen et al., 2017; Chen et al., 2016a). These baselines cover network propagation, graph-regularized matrix factorization, heterogeneous graph inference, inductive matrix completion, semi-supervised learning, ranking-based nearest-neighbor prediction, and within-between score strategies. Under the reported five-fold comparison setting, RLNSF-MDA achieved the highest AUC and AUPR values among all methods (Table 8). The AUC of RLNSF-MDA was 0.9194, exceeding KATZMDA (0.9012) and GRNMF (0.8944), while its AUPR was 0.9055, markedly higher than the strongest baseline AUPR of 0.2543 from GRNMF.

**TABLE 8:** Comparison with state-of-the-art miRNA–disease association predictors.

Method	AUC	AUPR	Accuracy	Precision	Recall	F1-score
KATZMDA	0.9012	0.2270	0.9577	0.3249	0.3442	0.3311
GRNMF	0.8944	0.2543	0.9574	0.3391	0.4210	0.3754
HGIMDA	0.7952	0.1804	0.9594	0.3218	0.3000	0.3105
IMCMDA	0.7687	0.1324	0.9456	0.2101	0.2853	0.2420
RLSMDA	0.8712	0.2069	0.9537	0.2905	0.3558	0.3188
RKNNMDA	0.7445	0.1679	0.9718	0.5387	0.5118	0.5249
WBSMDA	0.7116	0.0437	0.8918	0.0732	0.2144	0.1078
RLNSF-MDA	0.9194	0.9055	0.8525	0.8525	0.8525	0.8525

**FIGURE 6:**
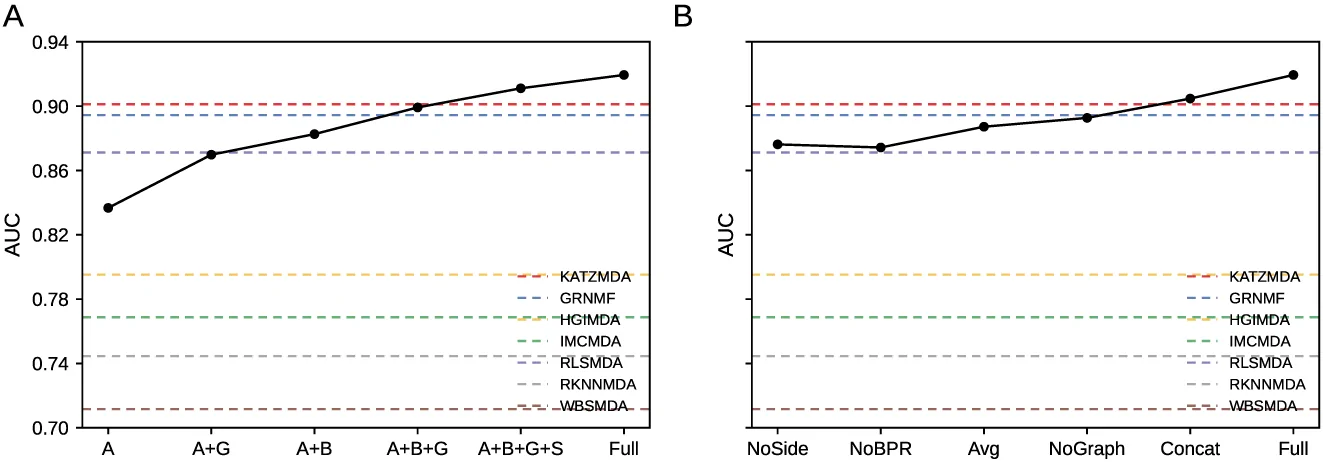
AUC comparison with state-of-the-art baselines and reduced RLNSF-MDA variants. **(A)** Comparison of the SOTA baseline methods with progressive feature combinations from the association-only setting to the full RLNSF-MDA model. **(B)** Comparison of the same baseline methods with fusion-strategy variants, including removal of side scores, removal of BPR loss, average fusion, removal of graph regularization, and concatenation of side features. A + B + G + S denotes association, biological similarity, GIP, and side-score features. Avg, average fusion; BioSim, biological similarity; BPR, Bayesian personalized ranking; GIP, Gaussian interaction profile; NoGraph, removal of graph regularization; NoSide, removal of side scores.

The comparison also showed that threshold-based accuracy alone was not sufficient to describe performance in this benchmark. RKNNMDA obtained the highest accuracy among the baselines (0.9718), but its AUC and AUPR were 0.7445 and 0.1679, respectively. In contrast, RLNSF-MDA achieved balanced values across AUC, AUPR, precision, recall, and F1-score, all of which were 0.8525 or higher, except that no separate MCC metric was reported in this table. Figure 6 visualizes the AUC comparison against the SOTA baselines and the internal feature/fusion variants, showing that the full RLNSF-MDA model remained above both the external baselines and reduced variants.

### 3.6 Immune-related case studies

To evaluate whether RLNSF-MDA could prioritize biologically traceable immune-related miRNAs, we performed case studies for lupus nephritis, lymphoma, and leukemia. Following the case-study presentation style used in MUSCLE, candidate miRNAs were listed by rank and then checked against external evidence from the exported miR2Disease and dbDEMC 2.0 case-study resources (Jiang et al., 2009; Yang et al., 2017). The summary file contained 45 confirmed miRNAs for lupus nephritis, 47 confirmed miRNAs for lymphoma, and 47 confirmed miRNAs for leukemia. Unconfirmed entries were retained at randomized positions in each ranked list as candidates for future experimental validation rather than counted as database-supported associations.

For lupus nephritis, 45 of the top 50 listed miRNAs had supporting evidence in the case-study summary (Table 9). The highest-ranked confirmed candidates included hsa-let-7a, hsa-let-7e, hsa-mir-124a, hsa-mir-130b, and hsa-mir-133a, all of which were linked to lupus nephritis evidence in miR2Disease. For lymphoma, 47 of the top 50 listed miRNAs were confirmed (Table 10). The first five confirmed candidates, hsa-mir-17, hsa-let-7a, hsa-mir-143, hsa-mir-155, and hsa-let-7c, were supported by lymphoma evidence from dbDEMC v2.0, miR2Disease, or both resources. For leukemia, 47 of the top 50 listed miRNAs were confirmed (Table 11). The highest-ranked confirmed candidates, including hsa-mir-223, hsa-mir-126, hsa-mir-130b, hsa-mir-21, and hsa-mir-221, had evidence of association with leukemia in dbDEMC v2.0 and miR2Disease. Together, these disease-specific tables show that the immune-related ranked lists recovered many database-supported miRNAs while still leaving a small set of unconfirmed candidates for follow-up.

**TABLE 9:** Top 50 candidate miRNAs for the lupus nephritis case study.

Rank	miRNA	Evidence status
1	hsa-let-7a	miR2Disease
2	hsa-let-7e	miR2Disease
3	hsa-mir-124a	miR2Disease
4	hsa-mir-130b	miR2Disease
5	hsa-mir-133a	miR2Disease
6	hsa-mir-134	miR2Disease
7	hsa-mir-3619	Unconfirmed
8	hsa-mir-142	miR2Disease
9	hsa-mir-150	miR2Disease
10	hsa-mir-15b	miR2Disease
11	hsa-mir-184	miR2Disease
12	hsa-mir-185	miR2Disease
13	hsa-mir-195	miR2Disease
14	hsa-mir-197	miR2Disease
15	hsa-mir-198	miR2Disease
16	hsa-mir-200c	miR2Disease
17	hsa-mir-208	miR2Disease
18	hsa-mir-196-2	Unconfirmed
19	hsa-mir-210	miR2Disease
20	hsa-mir-223	miR2Disease
21	hsa-mir-23a	miR2Disease
22	hsa-mir-296	miR2Disease
23	hsa-mir-30a	miR2Disease
24	hsa-mir-30d	miR2Disease
25	hsa-mir-320	miR2Disease
26	hsa-mir-324	miR2Disease
27	hsa-mir-325	miR2Disease
28	hsa-mir-345	miR2Disease
29	hsa-mir-346	miR2Disease
30	hsa-mir-365	miR2Disease
31	hsa-mir-149	Unconfirmed
32	hsa-mir-381	miR2Disease
33	hsa-mir-423	miR2Disease
34	hsa-mir-433	miR2Disease
35	hsa-mir-484	miR2Disease
36	hsa-mir-486	miR2Disease
37	hsa-mir-494	miR2Disease
38	hsa-mir-500	miR2Disease
39	hsa-mir-513	miR2Disease
40	hsa-mir-516	miR2Disease
41	hsa-mir-518b	miR2Disease
42	hsa-mir-3668	Unconfirmed
43	hsa-mir-518c	miR2Disease
44	hsa-mir-557	miR2Disease
45	hsa-mir-575	miR2Disease
46	hsa-mir-583	miR2Disease
47	hsa-mir-596	miR2Disease
48	hsa-mir-600	miR2Disease
49	hsa-mir-329	Unconfirmed
50	hsa-mir-601	miR2Disease

Confirmed candidates were checked against the case-study evidence file, and unconfirmed entries were retained for future validation.

**TABLE 10:** Top 50 candidate miRNAs for the lymphoma case study.

Rank	miRNA	Evidence status
1	hsa-mir-17	dbDEMC v2.0; miR2Disease
2	hsa-let-7a	dbDEMC v2.0; miR2Disease
3	hsa-mir-143	dbDEMC v2.0; miR2Disease
4	hsa-mir-155	dbDEMC v2.0; miR2Disease
5	hsa-let-7c	dbDEMC v2.0; miR2Disease
6	hsa-mir-139	dbDEMC v2.0; miR2Disease
7	hsa-mir-150	dbDEMC v2.0; miR2Disease
8	hsa-mir-199a	dbDEMC v2.0; miR2Disease
9	hsa-mir-21	dbDEMC v2.0; miR2Disease
10	hsa-mir-26a	dbDEMC v2.0; miR2Disease
11	hsa-mir-6747	Unconfirmed
12	hsa-mir-30a	dbDEMC v2.0; miR2Disease
13	hsa-let-7e	dbDEMC v2.0; miR2Disease
14	hsa-let-7g	dbDEMC v2.0
15	hsa-mir-106a	dbDEMC v2.0; miR2Disease
16	hsa-mir-142	dbDEMC v2.0; miR2Disease
17	hsa-mir-210	dbDEMC v2.0; miR2Disease
18	hsa-mir-26b	dbDEMC v2.0
19	hsa-mir-29c	dbDEMC v2.0; miR2Disease
20	hsa-mir-342	dbDEMC v2.0
21	hsa-mir-9	dbDEMC v2.0; miR2Disease
22	hsa-mir-96	dbDEMC v2.0; miR2Disease
23	hsa-mir-99a	dbDEMC v2.0; miR2Disease
24	hsa-mir-107	dbDEMC v2.0; miR2Disease
25	hsa-mir-126	dbDEMC v2.0
26	hsa-mir-130a	dbDEMC v2.0; miR2Disease
27	hsa-mir-4755	Unconfirmed
28	hsa-mir-198	dbDEMC v2.0; miR2Disease
29	hsa-mir-199b	dbDEMC v2.0; miR2Disease
30	hsa-mir-19a	dbDEMC v2.0; miR2Disease
31	hsa-mir-19b	dbDEMC v2.0; miR2Disease
32	hsa-mir-221	dbDEMC v2.0; miR2Disease
33	hsa-mir-222	dbDEMC v2.0; miR2Disease
34	hsa-mir-223	dbDEMC v2.0; miR2Disease
35	hsa-mir-34a	dbDEMC v2.0; miR2Disease
36	hsa-mir-424	dbDEMC v2.0
37	hsa-mir-93	dbDEMC v2.0; miR2Disease
38	hsa-mir-95	dbDEMC v2.0; miR2Disease
39	hsa-let-7f	dbDEMC v2.0; miR2Disease
40	hsa-mir-1	dbDEMC v2.0; miR2Disease
41	hsa-mir-132	dbDEMC v2.0; miR2Disease
42	hsa-mir-134	dbDEMC v2.0
43	hsa-mir-138	dbDEMC v2.0; miR2Disease
44	hsa-mir-5584	Unconfirmed
45	hsa-mir-145	dbDEMC v2.0; miR2Disease
46	hsa-mir-16	dbDEMC v2.0; miR2Disease
47	hsa-mir-181a	dbDEMC v2.0; miR2Disease
48	hsa-mir-192	dbDEMC v2.0; miR2Disease
49	hsa-mir-200a	dbDEMC v2.0; miR2Disease
50	hsa-mir-204	dbDEMC v2.0; miR2Disease

Confirmed candidates were checked against the case-study evidence file, and unconfirmed entries were retained for future validation.

**TABLE 11:** Top 50 candidate miRNAs for the leukemia case study.

Rank	miRNA	Evidence status
1	hsa-mir-223	dbDEMC v2.0; miR2Disease
2	hsa-mir-126	dbDEMC v2.0; miR2Disease
3	hsa-mir-130b	dbDEMC v2.0; miR2Disease
4	hsa-mir-21	dbDEMC v2.0; miR2Disease
5	hsa-mir-221	dbDEMC v2.0; miR2Disease
6	hsa-mir-93	dbDEMC v2.0; miR2Disease
7	hsa-mir-106a	dbDEMC v2.0; miR2Disease
8	hsa-mir-140	dbDEMC v2.0; miR2Disease
9	hsa-mir-548az	Unconfirmed
10	hsa-mir-155	dbDEMC v2.0; miR2Disease
11	hsa-mir-17	dbDEMC v2.0; miR2Disease
12	hsa-mir-181b	dbDEMC v2.0; miR2Disease
13	hsa-mir-181c	dbDEMC v2.0; miR2Disease
14	hsa-mir-192	dbDEMC v2.0; miR2Disease
15	hsa-mir-23a	dbDEMC v2.0; miR2Disease
16	hsa-mir-26b	dbDEMC v2.0
17	hsa-let-7i	dbDEMC v2.0
18	hsa-mir-106b	dbDEMC v2.0; miR2Disease
19	hsa-mir-107	dbDEMC v2.0; miR2Disease
20	hsa-mir-130a	dbDEMC v2.0; miR2Disease
21	hsa-mir-143	dbDEMC v2.0; miR2Disease
22	hsa-mir-150	dbDEMC v2.0; miR2Disease
23	hsa-mir-15a	dbDEMC v2.0; miR2Disease
24	hsa-mir-22	dbDEMC v2.0; miR2Disease
25	hsa-mir-222	dbDEMC v2.0; miR2Disease
26	hsa-mir-4535	unconfirmed
27	hsa-mir-23b	dbDEMC v2.0; miR2Disease
28	hsa-mir-424	dbDEMC v2.0; miR2Disease
29	hsa-let-7a	dbDEMC v2.0; miR2Disease
30	hsa-let-7c	dbDEMC v2.0; miR2Disease
31	hsa-let-7e	dbDEMC v2.0; miR2Disease
32	hsa-let-7g	dbDEMC v2.0
33	hsa-mir-128b	dbDEMC v2.0; miR2Disease
34	hsa-mir-151	dbDEMC v2.0; miR2Disease
35	hsa-mir-15b	dbDEMC v2.0; miR2Disease
36	hsa-mir-181a	dbDEMC v2.0; miR2Disease
37	hsa-mir-182	dbDEMC v2.0; miR2Disease
38	hsa-mir-6865	unconfirmed
39	hsa-mir-184	dbDEMC v2.0; miR2Disease
40	hsa-mir-195	dbDEMC v2.0; miR2Disease
41	hsa-mir-198	dbDEMC v2.0
42	hsa-mir-199b	dbDEMC v2.0; miR2Disease
43	hsa-mir-210	dbDEMC v2.0; miR2Disease
44	hsa-mir-27a	dbDEMC v2.0; miR2Disease
45	hsa-mir-28	dbDEMC v2.0
46	hsa-mir-29b	dbDEMC v2.0; miR2Disease
47	hsa-mir-29b-2	dbDEMC v2.0; miR2Disease
48	hsa-mir-29c	dbDEMC v2.0; miR2Disease
49	hsa-mir-30d	dbDEMC v2.0; miR2Disease
50	hsa-mir-30e	dbDEMC v2.0

Confirmed candidates were checked against the case-study evidence file, and unconfirmed entries were retained for future validation.

## Data Availability

The original contributions presented in the study are included in the article/supplementary material; further inquiries can be directed to the corresponding author.
